# Characterization of Leaf Rust Resistance in International Barley Germplasm Using Genome-Wide Association Studies

**DOI:** 10.3390/plants12040862

**Published:** 2023-02-14

**Authors:** Laura A. Ziems, Lovepreet Singh, Peter M. Dracatos, Mark J. Dieters, Miguel Sanchez-Garcia, Ahmed Amri, Ramesh Pal Singh Verma, Robert F. Park, Davinder Singh

**Affiliations:** 1 Faculty of Science, School of Life and Environmental Sciences, The University of Sydney, Sydney, NSW 2570, Australia; 2 Department of Animal, Plant and Soil Sciences, AgriBio, La Trobe University, Bundoora, VIC 3086, Australia; 3 School of Agriculture and Food Sciences, The University of Queensland, Brisbane, QLD 4067, Australia; 4 International Centre for Agriculture Research in Dry Areas (ICARDA), Rabat 10170, Morocco; 5 Indian Institute of Wheat and Barley Research, Karnal 132001, India

**Keywords:** adult-plant resistance, barley, genome-wide association studies, *Hordeum vulgare*, *Puccinia hordei*, leaf rust, *Rph*

## Abstract

A panel of 114 genetically diverse barley lines were assessed in the greenhouse and field for resistance to the pathogen *Puccinia hordei*, the causal agent of barley leaf rust. Multi-pathotype tests revealed that 16.6% of the lines carried the all-stage resistance (ASR) gene *Rph3*, followed by *Rph2* (4.4%), *Rph1* (1.7%), *Rph12* (1.7%) or *Rph19* (1.7%). Five lines (4.4%) were postulated to carry the gene combinations *Rph2+9.am*, *Rph2+19* and *Rph8+19*. Three lines (2.6%) were postulated to carry *Rph15* based on seedling rust tests and genotyping with a marker linked closely to this gene. Based on greenhouse seedling tests and adult-plant field tests, 84 genotypes (73.7%) were identified as carrying APR, and genotyping with molecular markers linked closely to three known APR genes (*Rph20*, *Rph23* and *Rph24*) revealed that 48 of the 84 genotypes (57.1%) likely carry novel (uncharacterized) sources of APR. Seven lines were found to carry known APR gene combinations (*Rph20+Rph23*, *Rph23+Rph24* and *Rph20+Rph24*), and these lines had higher levels of field resistance compared to those carrying each of these three APR genes singly. GWAS identified 12 putative QTLs; strongly associated markers located on chromosomes 1H, 2H, 3H, 5H and 7H. Of these, the QTL on chromosome 7H had the largest effect on resistance response to *P. hordei*. Overall, these studies detected several potentially novel genomic regions associated with resistance. The findings provide useful information for breeders to support the utilization of these sources of resistance to diversify resistance to leaf rust in barley and increase resistance durability.

## 1. Introduction

Cultivated barley is an important cereal crop that is ranked fourth in terms of global food production after wheat, maize and rice [1,2]. In Australia, it is the second most important food crop after wheat [3], adding billions of dollars to the national economy. Barley is used primarily for feed and malting purposes, and its production can be severely hampered by various diseases, including four that are caused by rust pathogens (crown rust, leaf/brown rust, stem rust and stripe/yellow rust). Among these diseases, leaf rust (BLR) caused by the fungal pathogen *Puccinia hordei* Oth. is the most common and widely distributed, occurring throughout the barley-growing regions of Africa, Asia, Australia, Europe, New Zealand, North America and South America [4,5]. Early infections of BLR can cause up to 32% yield losses in susceptible varieties in Australia and North America [6], with losses as high as 60% in very susceptible varieties [7,8].

In Australia, BLR has emerged as a significant national limitation to the production of superior-quality barley in all important barley-growing areas due to inadequate resistance; continued barley production in disease-prone, high-rainfall areas; and/or mutational changes that have rendered genetic resistance ineffective [9]. Approaches to controlling leaf rust include cultural practices, chemicals and the use of resistant varieties. Among these, genetic resistance is the the most economical, eco-friendly and sustainable strategy [10]. Nevertheless, the poor durability of single-gene resistance in many modern barley varieties in Australia [9] and worldwide necessitates further searches for and the introgression of new sources of resistance, especially those sources considered to provide durable resistance. Pyramiding multiple, diverse sources of resistance in new varieties to counter the continuous evolution of new virulent pathotypes within the pathogen population is considered the best approach to achieving durable resistance [11].

Based on plant growth stage, resistance is categorized broadly into two classes, i.e., ‘Seedling’ or ‘All-Stage Resistance’ (ASR) and ‘Adult-Plant Resistance’ (APR). ASR genes are easier to phenotype in the greenhouse and incorporate into breeding programs but are less durable because they are usually controlled by single major genes that are race-specific [12]. To date, 25 ASR genes conferring resistance to *P. hordei* (*Rph1*-*Rph19*, *Rph21*, *Rph22* and *Rph25-Rph28*) have been mapped and catalogued [13]; however, most of these have been rendered ineffective by the evolution of new pathotypes of *P. hordei*. APR is expressed at adult growth stages only and is often race-non-specific and durable [14]. To date, three APR genes—*Rph20* [15], *Rph23* [11] and *Rph24* [16]—conferring resistance to *P. hordei* have been reported and proven to provide durable resistance to BLR.

The ineffectiveness of most ASR genes and the limited diversity of APR to *P. hordei* emphasizes the importance of searching for and characterizing new sources of resistance to diversify the resistance available for use in controlling the disease. Multi-pathotype testing is the starting point in the genetic evaluation of rust resistance [17,18], allowing the characterization of germplasm for known rust resistance genes. The information generated through these tests can assist in identifying/developing gene combinations to enhance the durability of leaf rust resistance in new barley varieties. Molecular markers linked to rust resistance genes, where available, can further assist in the identification of resistance and the determination of whether they are different from those described previously. Very closely linked codominant markers are now available for all three APR genes [19], the ASR genes *Rph7* [20] and *Rph13* [21], and gene-based predictive markers have been developed for *Rph3* [22] and *Rph15* [23]. These markers are very useful for marker-assisted selection and validating new resistances. 

Genome-wide association mapping (GWAS) has become a common approach to characterizing the genetic architecture of a range of traits in animals and plants, especially when analysing large germplasm collections with historical data sets [14,24,25]. GWAS is a rapid approach that avoids the need to develop specific mapping populations (e.g., biparental crosses). GWAS identifies genomic regions associated with a trait, relying on historic linkage disequilibrium between observed characteristics and single-nucleotide polymorphisms (SNPs), which can be further refined through fine mapping approaches [14,24]. Several studies have applied GWAS in barley to identify genomic regions associated with resistance to leaf rust [14,16], stem rust [26] and stripe rust [27]. 

The International Center for Agriculture Research in Dry Areas (ICARDA) has the CGIAR global mandate to breed barley varieties for the developing world. As such, more than 250 spring and winter two-row, six-row and naked barley varieties of ICARDA origin have been released in 46 countries, 51 of them in the last 10 years. Besides direct releases, the impact of ICARDA germplasm in both the developed and the developing worlds’ breeding programs is widely recognized as parental material providing genetic diversity and new germplasm sources for traits of interest. The center holds in trust one of the largest barley collections in the world with the highest number of landraces and wild relative species. These resources are utilized by breeders within ICARDA and internationally to develop high-performing barley varieties in terms of yield, nutrition and resistance to various diseases and pests. To further utilize the barley genetic resources conserved and developed by ICARDA, a collaborative initiative called the CAIGE (CIMMYT Australia ICARDA Germplasm Evaluation) project (www.caigeproject.org.au; accessed on 25 February 2020) was launched. Under the CAIGE initiative, Australia receives new barley germplasm from ICARDA that is screened for yield potential, agronomic performance and resistance to a range of foliar diseases (including BLR). The germplasm and phenotypic information are made available to Australian breeders and researchers for the purposes of identifying novel traits and the introgression of desirable genomic regions into agronomically relevant material for release to growers. 

In this study, we evaluated and characterized a panel of 114 barley genotypes imported through the CAIGE program in 2018 for resistance to leaf rust. The aim of the study was to provide detailed information on BLR resistance to enhance and accelerate the utilization of the ICARDA germplasm to develop new barley varieties with durable resistance to leaf rust. An integrated strategy using multi-pathotype testing in the greenhouse, field screening with defined pathotypes, molecular-marker screening for known genes, and GWAS was applied to characterize this panel and identify putatively novel genomic regions associated with resistance to leaf rust.

## 2. Results

### 2.1. Greenhouse Seedling Tests

**Figure 1:**
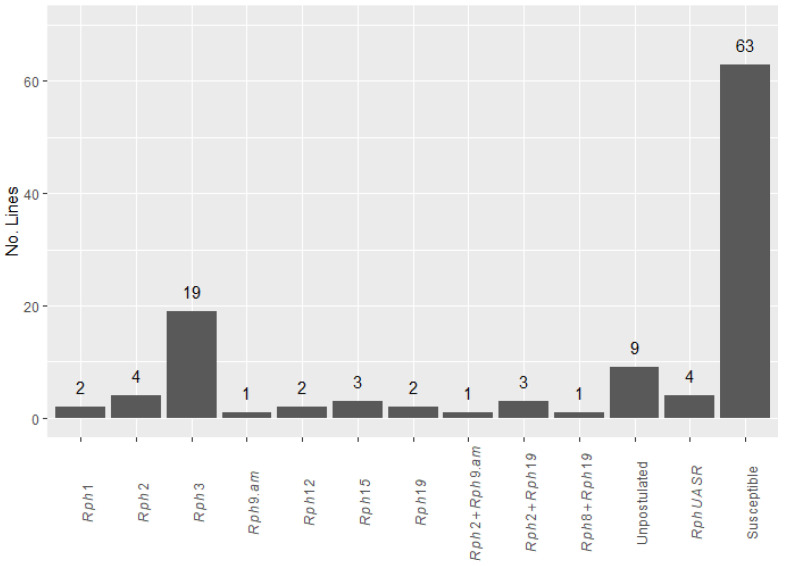
Frequency distribution of known and unknown *Rph* genes postulated in 114 CAIGE lines.

A range of infection-type (IT) responses was observed in the 114 lines screened with nine pathotypes of *P. hordei* in the greenhouse. The IT patterns shown by the test genotypes and differential genotypes were compared to postulate known seedling gene(s) and to identify any uncharacterized resistance gene(s) present (Figure 1; Table S1). Of the 114 test lines, 63 displayed a high IT response (33+ to 3+) to all pathotypes and were therefore concluded to lack seedling resistance genes that were effective against at least one of the pathotypes used in this study (viz., *Rph1*, *Rph2*, *Rph3*, *Rph4*, *Rph5*, *Rph7*, *Rph8*, *Rph9*, *Rph10*, *Rph11*, *Rph12*, *Rph13*, *Rph14*, *Rph15*, *Rph17*, *Rph18*, *Rph19*, *Rph21*, *Rph22*, *Rph25*, *Rph27* and *Rph28*). Seven lines showed low IT responses against all pathotypes used; the resistance carried by these lines could be conferred by either *Rph7* or *Rph15*, a combination of both, unknown ASR gene(s), or known gene combinations for which virulence was not present among the pathotypes used. Marker analysis revealed the absence of *Rph7* in all lines and the presence of *Rph15* in three of the seven lines. Nine genotypes displayed IT patterns that did not match any of the differential genotypes or any effective gene combination expected with these pathotypes; therefore, it was not possible to characterize the seedling resistance in these lines. Thirty-five lines carried *Rph1*, *Rph2*, *Rph3*, *Rph8*, *Rph9.am*, *Rph12* and *Rph19* either singly or in combination. The remaining nine lines were resistant to at least one pathotype, and the resistance present could not be identified with the set of pathotypes used. 

### 2.2. Variation in Field Disease Response

Variation in adult-plant leaf rust response was observed across the 114 lines (Table S1). In 2019, leaf rust infection was established well in the field, and there was an obvious progression in disease from the first reading (when the disease levels in the susceptible Gus check reached 80–90%; R1_2019) to the second reading (when the disease levels in the susceptible Gus check reached 100%; R2_2019); the two data sets showed correlation (r^2^ = 0.64). The 2021 reading (taken when the disease levels in the susceptible Gus check reached 100%) showed correlations of 0.63 with R1_2019 and 0.97 with R2_2019. Landraces (LAN and FIG) and high-input breeding lines (ZBS and ZIC) showed correlations between 2019 readings (r^2^ = 0.58 and 0.72, respectively); the 2021 landrace reading showed a correlation of 0.58 with R1_2019 and a correlation of 0.96 with R2_2019. The 2021 high-input breeding lines showed correlations of 0.79 with R1_2019 and 0.99 with R2_2019. Low-input breeding lines (ZBT) showed a correlation (r^2^ = 0.85) between 2019 readings, and the 2021 reading showed correlations of 0.81 with R1_2019 and 0.99 with R2_2019. The correlations indicated that R2_2019 and 2021 were highly correlated and that the low-input breeding lines were stable across assessments. 

### 2.3. Assessment of APR in the Field and Marker Analysis

One hundred and seven lines were susceptible to pathotype 5457 P+ at the seedling stage in the greenhouse. Of these, 23 lines were susceptible (CI > 75) and 84 were resistant (CI < 75) at adult-plant growth stages in the field (R2_2019) with the same pathotype. The level of APR in the 84 lines identified as field-resistant ranged from low to high. Screening of these 84 lines with molecular markers linked to *Rph20*, *Rph23* and *Rph24* revealed the presence of *Rph20* in five, *Rph23* in eight and *Rph24* in 16 lines. Seven lines with high levels of APR carried the combinations *Rph20+Rph23* (two lines), *Rph23+Rph24* (two lines) and *Rph20+Rph24* (three lines). The remaining 48 lines with APR lacked all three markers, indicating that the APR in these lines is distinct from *Rph20*, *Rph23* and *Rph24*, and therefore likely novel. The resistance of these lines ranged from TR to 70S (Table S1). Seven genotypes that were seedling-resistant to all pathotypes in the greenhouse (including 5457 P+) also showed high levels of resistance in the field. Based on marker genotyping, three of these lines carried *Rph24* and one line carried the combination of *Rph23* and *Rph24* in addition to *RphUASR* (uncharacterized all-stage resistance) or *Rph15*. The resistance in the remaining three lines was either due to uncharacterized ASR alone or uncharacterized ASR and uncharacterized APR.

### 2.4. GWAS of Leaf Rust Resistance at Adult Growth Stages

**Figure 2:**
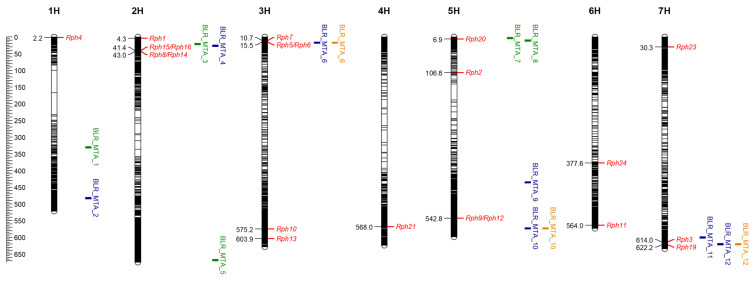
Visualization of known *Rph* genes in red (position based on BLAST search of published linked markers). QTLs identified at reading 1, 2019 represented in green, QTLs identified at reading 2, 2019 represented in blue and QTLs identified at the 2021 reading represented in orange, visualized on Morex v2 2019. QTLs were based on a single marker significantly associated at the 0.1% (−log10p > 3) level; a confidence interval of 2.5 Mbp was used for visualization purposes. Marker–trait associations (MTAs) represent genomic regions identified using genome-wide association mapping of breeding lines (high- and low-input) and landraces imported as part of the CAIGE program in 2018 and assessed in the field (Cobbitty NSW) at adult-plant stage in 2019 and 2021.

No clear genetic clustering of nursery type was observed (13.59% and 8.08% variance for PC1 and PC2, and 5.45% for PC3). Based on field data analysis, 12 putative QTLs were detected using the 9236 DArT-seq markers, with an average density of 2.16 markers/Mbp. Seven putative QTLs were co-located near the genomic positions of *Rph15/16/8/14*, *Rph7/5/6*, *Rph20* and *Rph3*/*19* (Table 1; Figure 2). Putative QTLs were detected on chromosomes 1H (MTA1, MTA2), 2H (MTA3, MTA4, MTA5), 3H (MTA6), 5H (MTA7, MTA8, MTA9, MTA10) and 7H (MTA11, MTA12). Of the putative QTLs identified, five (viz., MTA1, MTA3, MTA5, MTA7 and MTA8) were significant at the 0.1% level at R1_2019 (when susceptible Gus reached a disease severity of 90-100S), seven (viz., MTA2, MTA4, MTA6, MTA9, MTA10, MTA11 and MTA12) were significant at the 0.1% level at R2_2019 (seven days after the susceptible check reached DS 90-100S) and four (MTA6, MTA10, MTA11 and MTA12) were significant at the 0.1% level at 2021 and R2_2019. The significance of makers across assessments is indicated in Table 1.

**Figure 3:**
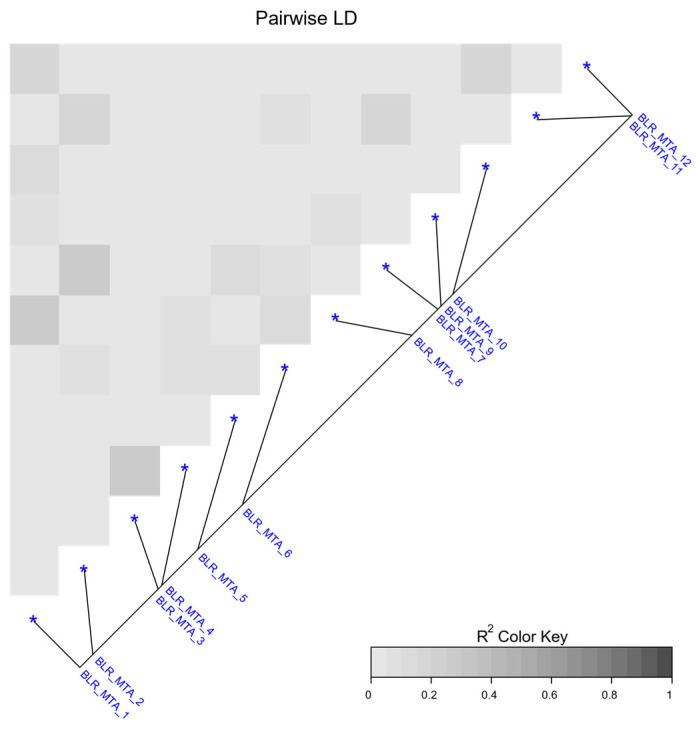
Linkage disequilibrium (LD) analysis of the 12 QTLs detected via GWAS performed on CAIGE lines. Heat maps represent pairwise LD as R^2^ between pairs of markers.

Of all the detected putative QTLs, MTA12, located on the long arm chromosome 7H, had the largest effect in controlling leaf rust. Marker 4792921 was the peak marker to this putative QTL at 620.61 Mbp, where the presence of the marker allele “A” at position 15 in the marker sequence was associated with resistance (Table 1). There was low LD observed across the QTLs (Figure 3). The strongest LD observed was between MTA3 and MTA4; the putative QTLs are located on the same chromosome (2H) within 5 Mbp (Table 1). LD was also observed between MTA_1 and MTA8, as well as MTA2 and MTA7, indicating a link between *Rph20* and genomic regions at the bottom of chromosome 1H. Five putative QTLs appear to be novel.

## 3. Discussion

For sustainable gene-based disease control, deployed resistance must be durable and diverse [5]. Therefore, it is extremely important to have a sound understanding of the resistance genes available in germplasm before it is exploited intensively in breeding programs [14]. To date, 25 ASR genes conferring resistance to *P. hordei* in barley have been catalogued [13], most of which have been overcome by adaptation of the pathogen [5]. Most ASR genes that remain effective are derived from wild relatives of barley (i.e., *Hordeum spontaneum* or *H. bulbosum*), and linkage drag can make them less attractive in barley breeding. Three APR genes, *Rph20* [15], *Rph23* [11] and *Rph24* [16], are considered to be pathotype-non-specific and provide low-to-moderate levels of protection individually and moderate-to-high levels in combination [5,11,16]. Robust, closely linked markers are available for these three genes [19], and consequently they are being targeted more as sources of leaf rust resistance by barley breeders. Although these three APR genes have been shown to be race-non-specific thus far, it should not be presumed that this will always be the case, as APR can be race-specific [28]. This emphasizes the need to characterize additional APR and ASR genes to enable the diversification of exploited resistance regions and the development of barley cultivars with durable leaf rust resistance. In this study, we targeted 114 diverse barley genotypes to identify and characterize known and new sources of resistance to *P. hordei* in barley for use in resistance breeding. 

Although multi-pathotype tests in this study detected several known ASR genes singly or in combination, more than 55% of the lines were susceptible to all the pathotypes tested. Out of the known seedling resistance genes detected, the most common was *Rph3* (in 19 lines; 16.6%), followed by *Rph2* (4.3%), *Rph1* (1.75%), *Rph12* (1.75%) and *Rph19* (1.75%). Five lines (4.4%) were postulated to carry the gene combinations *Rph2+9.am*, *Rph2+19* and *Rph8+19* (Table S1). Virulence to all these ASR genes has been reported in Australia [5], and therefore they have limited value. 

Three lines (34, 49 and 81; Table S1) were postulated to carry *Rph15* based on genotyping with a marker linked closely to *Rph15* [23]. Virulence for *Rph15* has not yet been detected in Australia and is extremely rare worldwide; only one isolate, 90-3, from Israel, has been reported to carry virulence for *Rph15* [29]. The rare occurrence of virulence for *Rph15* is most likely due to its limited use in agriculture [5], possibly because it originates from *H*. *spontaneum* and hence may have associated linkage drag. Although *Rph15* could possibly also be overcome if deployed singly in cultivars [30], it may play a valuable role if deployed in combination with other effective resistance genes. This gene was recently cloned and shown to encode a coiled-coil, nucleotide-binding, leucine-rich repeat (NLR) protein with an integrated Zinc-finger BED (ZF-BED) domain [29]. Based on sequence analysis, race specificity and the presence of a single-knockout mutant, the authors concluded that *Rph15* and *Rph16* are the same gene and not allelic, as previously suggested in several studies [31]. Of particular interest are four lines (23, 93, 99 and 114; Table S1) identified in our studies as carrying ASR effective against all pathotypes. These lines comprise one ICARDA elite breeding line (23; Soufara-02/3/RM1508/Por//WI2269/4/Hml-02/ArabiAbiad//ER/Apm/5/Arda/Moroc9-75), two landraces (93 and 99) collected near Herat (Afghanistan), and one landrace (114) collected near Krasnodar (Russia). These accessions lacked marker alleles associated with the *Rph7* or *Rph15* resistances, implying that the ASR (whether singly or in combination) in them is new and distinct from both *Rph7* and *Rph15* and therefore potentially very useful as a new source of resistance to *P. hordei*. It is probable that genotypes 93 and 99 carry the same gene because they were genetically similar in the GWAS analysis, and they were collected from the same area in Afghanistan. Genetic and allelic studies have been initiated to further characterize resistance in these four lines.

Unlike ASR genes with major effects, APR genes with minor or partial effects are considered less prone to being overcome by pathogen adaptation and hence durable [5]. Expression of APR relies on growth stage, environment and disease pressure [11,32], and the genes underlying APR are often additive, providing higher levels of resistance against *P.hordei* when combined [14]. Based on field testing, 84 genotypes were resistant at adult growth stages, and partitioning of these lines based on linked molecular markers revealed that 48 (57.1%) lacked any of the three known APR genes and hence are likely to be sources of novel, uncharacterized APR. Of these 48 lines, 36 were landraces or wild relatives. Up to 16 of these lines carrying unknown APR were from Ethiopia and the rest were from 12 other countries. This geographic diversity emphasizes the potential for novel sources of APR genes among the germplasm explored in this study. 

These lines also showed APR that ranged from low to high, indicating that there is likely more than one gene conferring the APR resistance observed. Genetic analysis is needed to further characterize, isolate and facilitate the use of the resistance discovered in these lines. 

Seven lines in this study carried known APR gene combinations (*Rph20+Rph23*, two lines; *Rph23+Rph24*, two lines; and *Rph20+Rph24*, three lines), all of which showed high levels of APR, in contrast to the lines carrying any of these three APR genes singly. These studies once again demonstrated that the APR genes *Rph20*, *Rph23* and *Rph24* are highly additive, as was established by Singh et al. [11,14]. Such interaction/additivity can play a pivotal role in achieving durable resistance to *P. hordei* in barley.

Genome-wide association studies conducted on a panel of 98 lines found 12 putative QTLs across the panel, of which 5 were detected at reading 1, 2019 (R1_2019), 7 were detected at reading 2, 2019 (2019_R2) and 4 were detected at the 2021 reading (2021), that co-located with QTLs detected at 2019_R2, while 5 appear to be novel. MTA1 and MTA2 were detected at positions 331.01 and 482.85 Mbp on chromosome 1H and shown to be in linkage disequilibrium with *Rph20*-linked MTA. Several previous studies have also reported markers linked to QTLs in the area between 388.13 and 505.86 Mbp [15,25,33,34]. The putative QTLs MTA3 and MTA4 are located in the same region as *Rph8/14/15/16* and are in linkage disequilibrium, which indicates that they are inherited together. Two QTLs (MTA3 and MTA4) were detected in the same region as that of *Rph8/14* and *Rph15/Rph16* [29,30]. As *Rph8/14* and *Rph15/16* are effective against the pathotype used in this study, the detection of these MTAs may be due to the contribution of resistance by these loci, considering that three lines in this study were found to carry *Rph15* based on linked markers [29]. *Rph15* is a major seedling resistance gene of strong effect; of three *Rph15/Rph16*-carrying lines, only two were included in the GWAS, and both were positive for alleles for resistance associated with MTA3 and MTA4.

MTA5 was identified on chromosome 2H at the position of 668.5 Mbp, in the vicinity of a QTL, Rphq2 (663.76 to 663.91 Mbp), reported by Qi et al. [35]. Several other studies [14,36,37,38] have also reported marker–trait associations in the same area between 631.51 and 667.72 Mbp. The relationships of the MTAs detected in our study to those reported previously require further validation.

MTA7 and MTA8 are located in the same genomic region as *Rph20* [15], and likely correspond to this gene. Putative APR QTLs were detected at R1_2019 only; R1 was timed to coincide with the susceptible genotype Gus reaching DS of 80-90S, and R2 was recorded seven days after this, when disease pressure was much higher. Disease pressure and other environmental factors, such as fluctuations in temperature, often lead to phenotypic variation in the field that prevents the accurate characterization of APR genes [32]. The minor APR gene *Rph20* becomes less effective under high disease pressure [11], likely accounting for MTAs corresponding to *Rph20* being detected at R1_2019 but not at other assessments. The linkage disequilibrium between these *Rph20*-linked markers and those at the distal end of 1H indicate that this region is enhanced by other minor genes, which is characteristic of APR.

This study was based on phenotypic data collected over two years from a single location; additional studies will facilitate the detection of additional minor-gene-based APRs expressed under different environmental pressures [11,32].

This study characterized leaf rust resistance in a set of diverse barley genotypes using integrated greenhouse multi-pathotype tests, field screening and the application of molecular markers. GWAS provided a valuable resolution in identifying marker–trait associations and genomic regions associated with leaf rust resistance that have been documented. Several sources of resistance appear to be potentially novel and should be further characterized for use in breeding and deployment in agriculture to diversify the genetic basis of resistance to leaf rust.

## 4. Materials and Methods

### 4.1. Plant Material

One hundred and fourteen barley genotypes/lines from the 2018 CAIGE project import identified as resistant to either BLR or other foliar diseases in Morocco were provided to the Plant Breeding Institute Cobbitty (PBIC) from the Australian Grain Genes (AGG) bank (Table S1). Of the 114 introduced lines, 78 were single-plant-selection landraces (identifier = LAN; passport data available in Table S2), 1 was a landrace selected for heat/drought tolerance (identifier = FIG), 22 belonged to the ICARDA high-input breeding program (identifiers = ZBS and ZIC) and 13 were from the ICARDA low-input program (identifier = ZBT). In addition, a set of 23 BLR differentials [5] were included in all disease-response screens as controls (Table 2). The set of differential genotypes is regularly purified and maintained at PBIC.

### 4.2. Pathogen Material 

Nine pathotypes of *P. hordei* (pts) were used in the greenhouse for seedling tests to postulate ASR *Rph* genes [9,39] (Table 2). Pathotype 5457 P+ was used in the field to assess adult-plant leaf rust response. Details of the virulence of these pathotypes with respect to known *Rph* genes and their culture numbers (i.e., genotypic identifiers) can be found in Table 3.

### 4.3. Greenhouse Screening

#### 4.3.1. Sowing and Plant Maintenance

Seeds of the test lines and differentials were sown in plastic pots containing a mixture of pine bark and coarse sand at a ratio of 4:1. The lines were sown as clumps in a clockwise direction, with three lines per pot (test lines) or five lines per pot (differentials), 10 seeds per line. The pots were fertilized with a soluble nitrogenous fertilizer Aquasol^®^ (Hortico Pty Ltd., Padstow NSW, Australia) at a rate of 30 g in 10 L of water for 200 pots, prior to sowing. After sowing, the pots were kept in a growth room maintained at 18 to 20 °C, where they were regularly watered and fertilized again one day prior to inoculation.

#### 4.3.2. Inoculation and Disease Assessment

Ten- to twelve-day old seedlings (with fully expanded first leaves) were inoculated and assessed for response to the nine pathotypes (Table 2). Urediniospores were mixed with mineral Isopar™ L (Exxon Mobil Corporation., Irving TX, USA) in a tube and misted with an atomizer over the top of seedlings in an inoculation chamber. The inoculated seedlings were transferred to an incubation room (without lights) for 18 to 24 h at an ambient temperature (18–24 °C), where artificial mist was generated by an ultrasonic humidifier. The incubated seedlings were then transferred to a microclimate greenhouse maintained at 22–24 °C. Disease response was assessed 10–12 days post-inoculation using the 0–4 infection type (IT) scale with cv. Gus as the susceptible control. ITs below 3 for the host indicated resistant responses and ITs of 3 or higher indicated susceptibility. IT response was further described using ‘−’ (less than average for the class), ‘+’ (more than average for the class), ‘C’ (chlorosis) and ‘N’ (necrosis); further details are provided by Park and Karakousi [6].

### 4.4. Field Screening 

Approximately 20–30 seeds from each line were packed in magazines for automated sowing in 0.7 m (metre) rows at 0.3 m spacings in the years 2019 and 2021 at PBIC. The leaf-rust-susceptible barley genotype ‘Gus’ was used as a disease spreader and was sown after every five plots of test lines for uniform disease infection. The field disease assessments were performed using a modified Cobb scale [41], based on a combination of disease severity (percentage of leaf area affected) and host response (R, no uredinia present; TR, trace or minute uredinia on leaves without sporulation; MR, small uredinia with slight sporulation; MR-MS, small-to-medium-sized uredinia with moderate sporulation; MS-S, medium-sized uredinia with moderate-to-heavy sporulation; S, large uredinia with abundant sporulation, uredinia often coalesced to form lesions).

The 2019 field disease assessments were performed at PBIC throughout disease progression in the month of October (weather station 94755099999: maximum temperature: 36 °C, minimum temperature: 4.3 °C, total precipitation: 8 mm, max daily precipitation: 5.5 mm, rain days: 9, max sustained wind: 44 kph [42]) when the disease levels on the susceptible Gus check reached 80–90% (reading 1; R1), followed by an assessment one week later when Gus was 100% susceptible (reading 2; R2). The 2021 field disease assessments were performed at PBIC in the month of October (weather station 94755099999: maximum temperature: 31 °C, minimum temperature: 6.6 °C, total precipitation: 93 mm, max daily precipitation: 23 mm, rain days: 18, max sustained wind: 41 kph [42]) when the disease levels on the susceptible Gus was 100% susceptible. An early reading could not be performed in this environment due to rain events washing away spores preventing accurate disease-response data collection.

The disease-severity data and host-response data were combined into a single value, the coefficient of infection (CI), by assigning a specific constant value of 0.1, 0.2, 0.4, 0.6, 0.8 and 1 to host-response ratings of TR, MR, MRMS, MS, MSS, and S, respectively, and multiplying by the percentage of leaf area affected. 

### 4.5. Molecular Marker Analysis for ASR and APR

#### 4.5.1. DNA Extraction

Three to four young leaves per test line were collected in an Eppendorf tube and kept over silica gel for 2–3 days for drying. Two ball bearings were added per tube to crush the dried leaves using a tissue lyser for 2 min at 20 rpm. Ball bearings were removed after crushing, and 800 μL of CTAB buffer was added to each tube. The samples were incubated for 30–40 min at 65 °C and then kept at room temperature for 5 min. In each sample tube, two layers formed upon adding a chloroform: phenol mixture (600 μL; 24:1 *v*:*v*). The two layers were mixed by inverting the tubes for 2 min, and then the samples were centrifuged at 10,000 rpm at 10 min. A quantity of 600 μL of supernatant was transferred to a 1.5 mL clean tube, followed by the addition of 500 μL cold isopropanol. Samples were kept at −20 °C for 20 min and then centrifuged at 10,000 rpm for 10 min. The supernatant was discarded, and the tubes were dried to remove all Ethanol. To wash the pellet of DNA, 500 μL washing buffer was added per tube and then centrifuged at 7000 rpm for 10 min. Samples were dried, 100 μL TE (pH 8) with RNAase (1 μL per 100 μL TE) was added to each tube, and they were kept in the oven at 37 °C for 1–2 h. The DNA samples were diluted to 50 ng/μL using double-distilled autoclaved water and quantified using a Nanodrop ND-1000 spectrophotometer (Nanodrop^®^ Technologies); the DNA stock was stored in a freezer at −20 °C until further use.

#### 4.5.2. Molecular Marker Screening

To check the presence (+) or absence (–) of APR genes (*Rph20*, *Rph23* and *Rph24*), three closely linked molecular markers were applied in all lines except those classified as susceptible. Lines with ASR response to all pathotypes at seedling growth stages were genotyped with molecular markers closely linked to *Rph7* and the co-dominant KASP marker within the *Rph15* gene. The primers were synthesized and supplied by Sigma; the sequence information of primers for each marker used is given in Table 4. To a quantity of 10 μL of PCR reaction mix was added 2 μL DNA, 2 μL 10 MiFi Buffer, 1μL of each forward and reverse primer (10 μM), 0.1 μL *taq* polymerase (Bioline), and 3.9 μL distilled autoclaved water. The PCR conditions used for the genotypic analysis using markers linked to *Rph15*-, *Rph20*-, *Rph23*- and *Rph24*-linked markers were previously described by Dracatos [23], Hickey et al. [15], Singh et al. [11] and Dracatos et al. [19], respectively. Alleles of different sizes were resolved using 2% agarose gel that was prepared by dissolving 2 g agarose per 100 mL of 1× Tris-borate EDTA (TBE) buffer (90 mM Tris-borate + 2 mM EDTA-pH 8.0), and 1 μL of GelRed was added per 100 mL of gel solution for staining. Each well was loaded with 2.5 μL PCR product, and a 100 bp ladder (Bioline) was used as a size reference. The positive DNA controls used for genotyping with markers linked to genes *Rph7*, *Rph15*, *Rph20*, *Rph23* and *Rph24* were the near-isogenic lines Bowman+*Rph7*, Bowman+*Rph15*, Flagship, Yerong and ND24260, respectively. Flagship was used as a negative control for markers linked to *Rph23* and *Rph24*, whilst Gus was used as a negative control for genotyping with the *Rph7*, *Rph15* and *Rph20* markers.

### 4.6. Phenotypic and Genotypic Data for GWAS

#### Filtering for Quality Markers and Genotypes

Genomic DNA was extracted from young tissue using a CTAB method described above and sent for DArT-Seq^TM^ genotyping under arrangements of the CAIGE project. DArT-Seq genotyping returned 63,473 polymorphic silico-DArT markers available at www.caigeproject.org.au/germplasm-evaluation-barley-genotypic-data (accessed on 1 December 2022). Poor-quality markers were removed through the following data curation: marker data were filtered for minor allele frequencies (MAFs) < 0.5%; those that failed to provide information for >20% of the lines were removed, and markers without a mapped position on the Barley Morex V2 genome assembly by TRITEX (2019) [43] were removed (16.9%). The resultant high-quality subset of 9236 markers was prepared for GWAS studies. GWAS was based on genotypes of 97 of the 114 individuals; 4 did not have both genotypic and phenotypic information available, 3 had > 20% missing genotypic values, and 10 were genetically indistinguishable. The individuals excluded from the GWAS are indicated in Table S1.

### 4.7. Population Structure Analysis Using PCA

**Figure 4:**
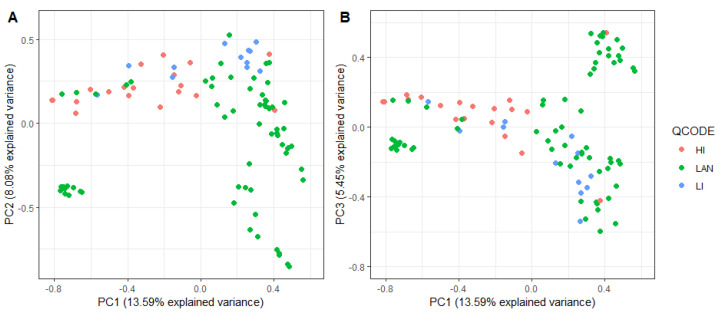
Principal component analysis of the kinship matrix visualizing the genetic relationships between 98 lines. The figure on the left (**A**) represents the first principal component (PC1; x-axis) and the second principal component (PC2; y-axis), and the figure on the right (**B**) represents PC1 (x-axis) and the third principal component (PC3; y-axis). In both plots, genotypes are coloured according to nursery type. LAN, landraces; HI, high-input breeding line (ZBS + ZIC); LI, low-input breeding line (ZBT).

Genetic relationships among accessions were investigated graphically based on DArT-Seq genotypes. Principal component analysis (PCA) was performed in R [44] using the “synbreed” package [45] to produce a similarity matrix. To compare genetic variation in the population, the PCA was drawn as a biplot using “ggplot 2” [46]. The first three principal components were depicted to visually determine whether individuals clustered according to nursery type (Figure 4). 

### 4.8. Genome-Wide Association Studies of Leaf Rust Resistance

**Figure 5:**
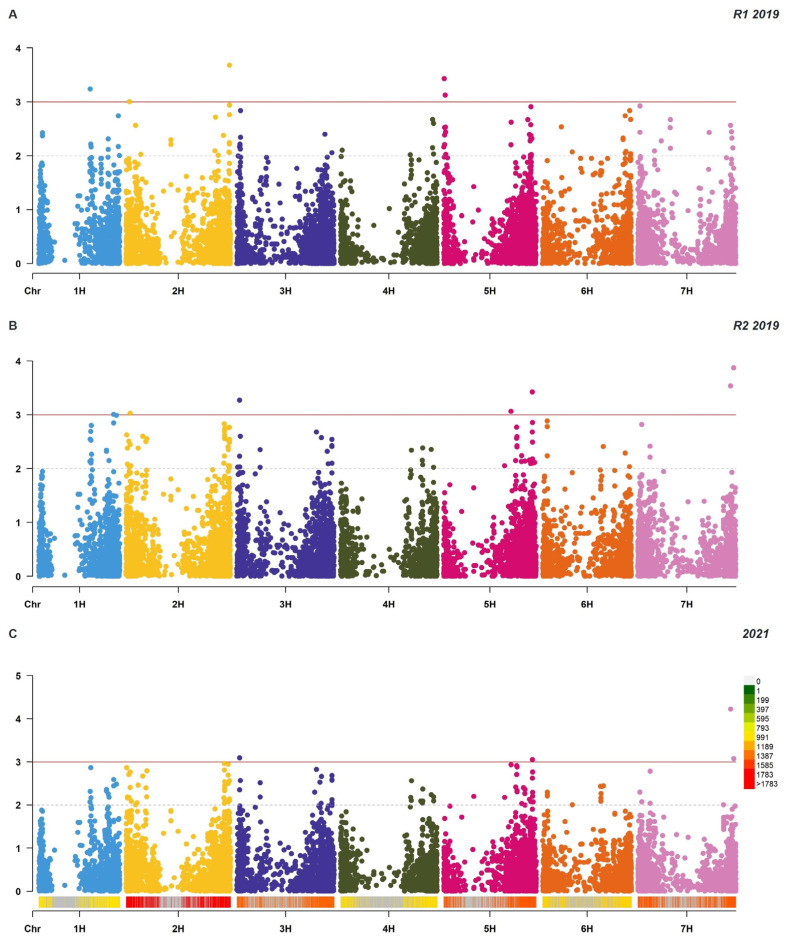
Manhattan plots representing markers associated with BLR resistance in a diverse set of barley CAIGE germplasms at three time points: reading 1, 2019 (**A**) reading 2, 2019 (**B**) and 2021 (**C**). The grey horizontal line represents a genome-wide significance threshold of −log10(p) of 2 (>1%), and the solid red horizontal line represents a genome-wide significance threshold of −log10(p) of 3 (>0.1%). Density bands colour-coded to show genotypic distribution of 9236 SNPs on chromosomes.

Kinship was estimated with genotypic similarity matrices formed using the “synbreed” package [45]. Genome-wide association analysis was conducted using the “rrBLUP” package [47] with 9236 markers and CIs (described previously) at two 2019 time points (2019_R1 and 2019_R2) and a single 2021 time point (2021). Significant marker–trait associations were determined using the threshold −log10p ≥ 3 (Figure 5). Twelve putative QTLs were detected and mapped on the Barley Morex V2 genome assembly by TRITEX (2019) [43]; known *Rph* gene positions were estimated using BLAST with markers reported as linked in previous studies and visualized with Mapchart 2.32 [48]. Markers associated with disease response were referred to as MTAs (marker–trait associations) or putative QTLs. Marker effects were calculated across all assessments for significant markers identified using modified the GWAS function in the “rrBLUP” package [47]. The allele for resistance for each putative QTL was determined from marker effects. Linkage disequilibrium (LD) analysis of the 12 putative QTLs was performed in R [44], an SNP matrix was calculated using the “chopsticks” package [49] and pairwise LD as r^2^ between pairs of markers was visualized using “LDheatmap” [50].

## Figures and Tables

**Table 1:** Summary of the marker–trait associations (MTAs) detected at three time points (R1_2019, R2_2019 and 2021) at adult-plant stage in field.

MTA Name	Marker ID	Chr ^1^	Position (Mbp) ^2^	Co-Located *Rph* Gene ^3^	R1_2019 ^4^	R2_2019 ^4^	2021 ^4^	SNP Position in Marker Sequence	Allele Resistant	Allele Susceptible
MTA_1	3258774	1H	331.01		34.16 ***	33.57 **	31.92 **	8	G	A
MTA_2	3262665	1H	482.85		NS ^5^	37.12 ***	34.01 **	7	G	A
MTA_3	3929195	2H	21.47	*Rph15/16/8/14*	29.41 ***	28.37 **	25.18 *	33	C	T
MTA_4	3256022	2H	26.34	*Rph15/16/8/14*	NS ^5^	31.70 ***	30.10 **	8	T	C
MTA_5	3261067	2H	668.5		32.93 ***	29.26 **	26.47 **	67	A	G
MTA_6	3266799	3H	17.35	*Rph7/5/6*	19.86 **	28.74 ***	28.02 ***	9	G	C
MTA_7	3434139	5H	4.05	*Rph20*	32.05 ***	NS ^5^	NS ^5^	61	C	T
MTA_8	5249701	5H	11.35	*Rph20*	31.10 ***	NS ^5^	NS ^5^	49	T	C
MTA_9	3396786	5H	436.05		21.49 **	28.48 ***	27.61 **	55	T	G
MTA_10	3256468	5H	573.69		NS ^5^	39.35 ***	37.17 ***	67	C	G
MTA_11	3260244	7H	600.48	*Rph3*	29.31 **	38.98 ***	43.01 ****	61	A	G
MTA_12	4792921	7H	620.61	*Rph3/19*	28.24 *	49.06 ***	43.47 ***	15	A	C

^1^ Chromosome; ^2^ Morex_rev2_2019; ^3^ MTA within 20 Mbp of known *Rph* gene considered co-located; ^4^ Effect of allele on phenotype; ^5^ NS—No significant association, * Significant at 5.0% level, ** Significant at 1.0% level, *** Significant at 0.1% level, **** Significant at 0.01% level—*p*-values derived from the genome-wide association model with four principal components.

**Table 2:** Infection-type ^1^ responses of differential genotypes with nine pathotypes of *Puccinia hordei*, based on which *Rph* genes were postulated.

Differential Genotype	*Rph* Gene	200 P−	220 P+ +*Rph13*	253 P−	4610 P+	5610 P+	5453 P+	5656 P+	5457 P−	5457 P+
Sudan	*Rph1*	−CN	0	3+	N	−N	3+	1−CN	3+	3+
Peruvian	*Rph2*	N	N	3−	1+CN	1+CN	3+	3+	3+C	3+C
Estate	*Rph3*	C	0	0	N	-CN	1−	3+	3+C	3+C
Gold	*Rph4*	1-	33+	3+	33+	3C	3+	3+	33+C	33+C
Magnif 104	*Rph5*	1−N	33+	N	N	-N	N	1−N	N	N
Bolivia	*Rph2+Rph6*	N	N	3+	CN	CN	33+	12-	3+	3+
Cebada Capa	*Rph7*	N	0	N	CN	-N	+N	1−CN	+CN	+N
Egypt 4	*Rph8*	33+	33+	33+	3+	33+	3+	12-CN	3+	3+
Abyssinian	*Rph9*	1N	N	3+	1CN	1+CN	33+	33+	3+	3+
Clipper BC8	*Rph10*	3+	33+	1+CN	1+CN	3+	3+	33+	33+	3+
Clipper BC67	*Rph11*	12+	1CN	12CN	2+C	2++C	3+	12−CN	33+	33+
Triumph	*Rph12*	+N	+N	1CN	33+C	3	3+	33+	3+	3+
PI 531849	*Rph13*	0	3+	−N	N	-N	CN	12−CN	+CN	+CN
PI 584760	*Rph14*	12+	1CN	1+CN	12C	12+CN	1+CN	33+	1CN	1+CN
Prior	*Rph19*	1	33+	1−CN	3+	3+	3+	33+	12−	3+
Cantala	*Rph9.am*	3+	3+	12−C	3+	3+	3+	3+	3+C	3+

^1^ Infection type (IT) based on a 0–4 scale [6]. ITs below 3 in the host indicated resistant responses and ITs of 3 or higher indicated susceptibility. IT response was further described using ‘−’ (less than average for the class), ‘+’ (more than average for the class), ‘C’ (chlorosis) and ‘N’ (necrosis).

**Table 3:** Pathotypes of *Puccinia hordei* used in the present study and their virulence profiles with respect to specific *Rph* genes.

Pathotype (Culture No.)	Virulence ^1^
200 P− (518) ^2^	*Rph8*
220 P+ +*Rph13* (577) ^2^	*Rph5*, *Rph8*, *Rph13*
253 P− (490)	*Rph1*, *Rph2*, *Rph4*, *Rph6*, *Rph8*
4610 P+ (491) ^2^	*Rph4*, *Rph8*, *Rph9*, *Rph12*
5610 P+ (520) ^2^	*Rph4*, *Rph8*, *Rph9*, *Rph10*, *Rph12*
5453 P+ (584)	*Rph1*, *Rph2*, *Rph4*, *Rph6*, *Rph9*, *Rph10*, *Rph12*, *Rph19*
5656 P+ (623)	*Rph2*, *Rph3*, *Rph4*, *Rph6*, *Rph8*, *Rph9*, *Rph12*, *Rph19*
5457 P− (626)	*Rph1*, *Rph2*, *Rph3*, *Rph4*, *Rph6*, *Rph9*, *Rph10*, *Rph12*
5457 P+ (612)	*Rph1*, *Rph2*, *Rph3*, *Rph4*, *Rph6*, *Rph9*, *Rph10*, *Rph12*, *Rph19*

^1^ Pathotype designation is based on the virulence pattern of an isolate on the differential set (*Rph1* to *Rph12*), using an octal notation system proposed by Gilmour [40]. The symbols ‘P−‘ and ‘P+’ denote avirulence and virulence, respectively, on genotype Prior (P) carrying *Rph19* [9]; ^2^ The pathogenicity of these pathotypes for *Rph6* is unknown due to avirulence on *Rph2* in each and the presence of this gene in the *Rph6* differential tester Bolivia (*Rph2+6*).

**Table 4:** Details of markers and sequences of the *Rph7*, *Rph15*, *Rph20*, *Rph23* and *Rph24* primers used in this study.

Gene	Marker	Chr ^1^	Forward Primer Sequence, 5′-3′	Reverse primer Sequence, 5′-3′	Reference
*Rph7*	Unknown	3H	GAGATAAAAGCATTACCAAAGGCTCAT	GCGCGCGCAACAGCAAACGGC	[20]
*Rph15*	Unknown	2H	TGAAGAAGCTGGAAGGTCACC	AGCCAAAAACCCTTCTGGCT	[23]
*Rph20*	*bPb0837*	5H	GACACTTCGTGCCAGTTTG	CCTCCCTCCCTCTTCTCAAC	[15]
*Rph23*	*Ebmac0603*	7H	ACCGAAACTAAATGAACTACTTCG	TGCAAACTGTGCTATTAAGGG	[11]
*Rph24*	*Sun43-44*	6H	CTAGACACCACCACCACACC	ATACCAGAGTTTGCGTCCGG	[19]

^1^ Chromosome.

## Data Availability

Publicly available genotypic datasets analyzed in this study can be found here: www.caigeproject.org.au/germplasm-evaluation-barley-genotypic-data/ (accessed on 25 February 2020). Phenotypic and molecular data presented in this study are available in Table S1. Passport data of landrace accessions presented in this study are available in Table S2.

## Supplementary Materials

The following supporting information can be downloaded at: https://www.mdpi.com/article/10.3390/plants12040862/s1, Table S1: Details of 114 CAIGE barley lines screened for leaf rust, and summary of phenotypic and molecular data based on greenhouse tests, field screening and marker analysis; Table S2: Passport data of the 78 CAIGE barley landrace accessions in this study.
